# 

**Published:** 1837-04-01

**Authors:** 


### BIBLIOGRAPHICAL RECORD;

OR,

*Works received for Review since last Quarter.*

1. Medical Essays. By J. Hungerford Sealy, M.D. No. 1, Phthisis Pulmonalis, its history and varieties, &c. Small 8vo, pp. 82. 1837.

---

2. A practical Treatise on the principal Diseases of the Lungs, considered especially in relation to the particular Tissues affected. By Geo. Hume Weatherhead, M.D. &c. Octavo, pp. 184, with a coloured plate. Churchill, February 1827.

---

3. The Pharmacopœia Collegii Regalis Medicorum Londinensis. Translated, with a Commentary, chemical, pharmaceutical, and medicinal, by D. Spillan, M.D. Fellow of the King and Queen's College of Physicians in Ireland. Pp. 305. Henry Renshaw, 1837.

---

4. A Translation of the Pharmacopœia of the Royal College of Physicians of London, 1836. With Notes and Illustrations. By Richard Phillips, F.R.S. L. and E., Lecturer on Chemistry at St. Thomas's Hospital. By permission. Octavo, pp. 392. S. Highley, 1837. Price 10*s*. 6*d*.

---

5. Conspectus Medicinæ Theoreticæ ad Versum Academicum. Auctore Jacobo Gregory, M.D. Olim. Med. Theor. nuper. Med. Pract. in Acad. Edin. Prof. Pars prima—Physiologiam et Pathologiam Complectens. Editio nova. Square 8vo, pp. 85. Sherwood, Gilbert, and Piper, 1837.

---

6. Interlineal Translation of Celsus de Medicinâ; with Ordo and Text. First four Books. By Robert Venables, A.M. M.B. Oxon. Second Ed. pp. 303. Sherwood, Gilbert, and Piper, 1837. Price 10*s*. 6*d*.

---

7. A Treatise on Painful and Nervous Diseases, and on a New Mode of Treatment for Diseases of the Eye and Ear. By A. Turnbull, M.D. Third edition, 8vo, pp. 161. John Churchill, 1837. Price 6*s*.

---

8. Outlines of Human Physiology. By Herbert Mayo, F.R.S. Senior Surgeon of the Middlesex Hospital, &c. Fourth edition, octavo, pp. 532. Henry Renshaw and J. Churchill, 1837.

---

9. First Principles of Surgery; being an Outline of Inflammation and its Effects. By George T. Morgan, A.M. Lecturer on Surgery in Aberdeen. Part I. Octavo, pp. 210. London: S. Highley, Fleet-st.—Edinburgh: Machlaclan & Stewart, 1837. Price 5*s*.

---

10. The Works of John Hunter, F.R.S., with Notes. Edited by Jas. F. Palmer, Senior Surgeon to the St. George's and St. James's Dispensary, &c. In Four Volumes. Vol. I. 8vo, pp. 632. Longman, Rees, and Co. Price 17*s*. 6*d*.

---

11. Sequel to Sematology; being an Attempt to clear the way for the Regeneration of Metaphysics. By the Author of "An Outline of Sematology, or an Essay towards establishing a new Theory of Grammar, Logic and Rhetoric." Octavo, pp. 192. 1837.

---

12. The practical Anatomy and elementary Physiology of the Nervous System; designed for the Use of Students in the Dissecting-room. By F. Le Gros Clark, Demonstrator of Anatomy in St. Thomas's Hospital. Octavo, pp. 367. Longman and Co. 1836.

---

13. Illustrations of the Comparative Anatomy of the Nervous System. By Joseph Swan. Part II. price 17*s*. Longman and Co. 1836.

---

14. Sketch of the Comparative Anatomy of the Nervous System; with Remarks on its Development in the Human Embryo: with Plates. To which is added, an arranged Descriptive List of the Series of illustrative Preparations and Drawings, and an Anatomical Table of the Analogies existing between the Brain of the Human Fœtus and that of the Lower Animals. By John Anderson, M.E.S. Sherwood, Gilbert, and Piper, 1837. Price 8s.

15. Nouveau Système de Physiologie Végétale et de Botanique, fondé sur les Méthodes d'Observations qui ont été developpées dans le nouveau Système de Chemie Organique. Par F. V. RASPAIL. Atlas 4to. Paris, chez J. B. Baillière, 1837.

---

16. A clinical Treatise on the Endemic Fevers of the West Indies. By W. J. EVANS. Octavo, 9s.

---

17. Lectures on Local Nervous Affections. By Sir B. C. BRODIE, Bart. Octavo, 4s.

---

18. Practical Observations on the Venereal Disease, and on the Use of Mercury. By ABRAHAM COLLES, M.D. one of the Surgeons of Dr. Stevens's Hospital. Octavo, pp. 351. Sherwood, Gilbert, and Piper, 1837. Price 9*s*.

---

19. Memoranda for young Practitioners in Midwifery. By E. RIGBY, M.D. &c. Lecturer on Midwifery at St. Thomas's Hospital, Assistant Physician to the General Lying-in Hospital. 12mo, pp. 48. Henry Renshaw, 1837.

---

20. Digest of the Homœopathic Principles. By Dr. EDWARD WILLIAMS, M.B. Cantab. Octavo, pp. 42. Hen. Renshaw, 1837.

---

21. Observations on the Preservation of Hearing, and on the choice, use, and abuse of Hearing Trumpets, &c. Fifth edition. By JOHN HARRISON CURTIS, Esq. M.R.I. Aurist and Occulist. Pp. 56. H. Renshaw, 1837.

---

22. An Essay on Insanity. Translated from the Author's Inaugural Dissertation composed on that subject, and submitted to the Faculty of Medicine in Edinburgh, preparatory to receiving the Degree of M.D. in 1833. By SAM. G. BAKEWELL, M.D. 2nd edition, 8vo, pp. 40 E. Cox, 1837. Price 1s.

23. J. PATERSON CLARK'S Treatise on the Teeth and Dentism. Square 12mo. 10*s*.

---

24. The Nervous System of the Human Body. By Sir C. BELL. Third edition, 8vo. 24s.

---

25. A Series of Anatomical Plates in Lithography, with References and Physiological Comments, &c. Edited by J. QUAIN, M.D. and WM. T. E. WILSON. Division II. Veins Fasciculi 45 and 46. February and March, 1837.

---

26. A History of British Quadrupeds. By THOMAS BELL, F.R.S. Lecturer on Zoology at King's College. Part 8, price 2*s*. 6*d*. illustrated with Wood-cuts and numerous Vignettes. 1837.

---

27. Practical Observations on Palpitation of the Heart. By J. C. WILLIAMS, M.D. Octavo, 6s.

---

28. Elements of Medicine. Vol. I. On Morbid Poisons. By RT. WILLIAMS, M.D. Octavo, pp. 338.

☞ *The Review of this important work is in type, but unavoidably deferred till next number.*

---

29. The Continental and British Medical Review, or Monthly Therapeutical Journal. Edited by Dr. BUREAUD RIOFREY. March 1, 1837.

☞ *In Exchange.*

---

### NOTICE TO AUTHORS.

Unfortunately our Bibliographical Record was lost about the middle of last Quarter, and several publications are not entered in that which is now published. We request those Authors who have sent us works not entered in the present number of the Journal, to furnish us with the title-pages of the omitted volumes.

### INDEX.

Abdomen, dropsy of the ........... 18
Aberdeen Lunatic Asylum........... 265
Abdominal pathology, illustrations of, 527
Abdominal parietes, abscess in ...... 528
Abortion of an undecomposed fœtus.. 187
Abscess in the liver, cases of........ 527
Acephalocystis .................... 326
Acute rheumatism, fatal case of ..... 510
Addison on fatty degeneration of liver 248
Advantages of statistical hosp. returns 262
Affection of the respiration ........ 314
Affection of the urinary organs...... 317
Affection of the digestive organs .... 319
Affection of the sensorium.......... 319
Affections of the nerves of sensation, 313
Age, influences of, on mortality...... 345
Aix ..... ........................ 85
Alcock, Dr. on the glosso-pharyngeal, 474
Amenorrhœa ...................... 116
Amputation for tetanus ............ 70
Amputation of the penis....... 178, 487
Analogous tissues ................ 333
Anasarca, causes of ............... 2
Anasarca, curious case of .......... 5
Anasarca, digitalis a remedy for .... 8
Anasarca, after scarlet fever ........ 14
Anasarca, with disease of the kidney.. 16
Anatomical knowledge, diffusion of .. 145
Anatomical engravings by Quain. .. 158
Anatomical remarks on hunchbacks.. 470
Anatomy of the serous membranes, Dr. Hodgkin on ................. 40
Anatomy and Physiology, Cyclopæd. of 158
Anatomy, recent publications on .... 451
Anatomy, recent researches in ...... 457
Anatomy of the tongue ... ......... 474
Anatomy, lectures on, as they were.. 489
Anatomy of the serous membranes .. 297
Andral's Clinique Medicale ........ 40
Aneurysm of the arteria innominata.. 282
Angina pectoris, nature of.......... 522
Angina pectoris, cause of .......... 523
Annan's new truss for prolapsus uteri 177
Anus, artificial, instrument for cure of 179
Apoplexy and phrenology .......... 225
Apoplexy.......................... 359
Apoplexy, Dr. Kennedy on ......... 479
Army, medical statistics of the.. 146, 269
Army, influence of foreign stations on 349
Arteria innominata, aneurysm of the.. 282
Arterial distribution of the spleen.... 531
Arteries, Knox's engravings of the .. 158
Ascites.......................... 18
Ascites, disease of the liver a cause of, 18
Ascites, arising from disease of heart, 20
Ascites, from disease of peritoneum.. 22
Asphyxia from foul air ............ 537
Asthma in infants ................. 520
Auscultation ...................... 104
Baden ........................... 85
Baden Baden ...................... 88
Bagnieres de Luction .. ............ 86
Balbirnie on the speculum ......... 105
Bareges .......................... 85
Baryta, poisoning by ........ ..... 205
Barytes, muriate of ............... 484
Basis, fracture of the .............. 573
Bichat, his system ................ 390
Bird's case of cystine .............. 256
Blood, mode of analyzing the ...... 221
Bloodvessels, formation of.......... 335
Bloxam, Mr. his case of inversio uteri, 169
Bonnet sur les maladies du foie ..... 124
Bony tumor of the face, case of ..... 565
Bostock, Dr. his erratum .......... 289
Bottle-nose, mode of paring a ...... 541
Bouillaud on medical philosophy .... 386
Bowels, spontaneous perforation of the 530
Brachial artery, false aneurysm of the, 485
Brain, Mr. Solly on the........ 158, 297
Brain, case of severe injury of ...... 202
Brain of the negro ................ 467
Brain, composition of the .......... 468
Brain, ulceration of the ........... 482
Bright on jaundice ................ 251
Bright on disease of the liver........ 553
British Medical Almanack .......... 156
British empire, statistics of the...... 345
British legion, hospitals of.......... 574
British Med. Association, laws of .... 587
Brodie, Sir B. on path. of spinal cord, 297
Brodie, Sir B. on nervous affections.. 463
Bunion ........................... 245
Bursa, enlarged, over the patella .... 242
Cæcum, Mr. Johnson on ulceration of, 197
Caries of the cervical vertebræ ...... 316
Carotids, Sir A. Cooper on tying the, 180
Carswell, Dr. on disease............ 297
Cat, thyroid gland of the ........... 460
Catalepsy, case of ................ 233
Cattle killed by lightning, post-mortem appearances in ................ 238
Cause of tropical fevers ............ 370
Cause of respirat. in new-born infants, 525
Causes of tetanus ................ 62
Cazeneuve on endocarditis ........ 513
Cellular tissue..................... 338
Cerebral apoplexy ................ 479
Cerebral symptoms................ 552
Cerebro-spinal irritation ........... 168
Chalybeates ...................... 86
Changes in the head .............. 488
Charcoal, astonishing effects of...... 143
Charter of the London University.... 282

Chemical properties of the secretions in health and in disease .......... 218
Children, on the management of .... 362
Children, physical education of..... 362
Children, mental cultivation ....... ... 363
Chili, state of medicine in .......... 203
Cholera at Naples ................. 505
Chomel on intermittent fevers ...... 506
Churchill on the umbilical cord..... 543
Clinical lectures of Dr. Graves ...... 550
Clinique medicale, by G. Andral .... 40
Cockburn on cerebro-spinal irritation, 168
Colchicum, poisoning from ........ 239
Colchicum, effects of .............. 240
College of Surgeons, ordinance of the, on the recognition of teachers .... 285
Competition, on the principles of... 579
Composition of the brain .......... 468
Composition of the spinal cord...... 468
Compound fracture of the skull .... 572
Congenital displacement of hip-joint, 36
Consumption a remittent fever ...... 136
Contagious typhus, Dr. Perry on .... 481
Contractility, two forms of ........ 390
Cooper, Sir A. on tying the carotids.. 180
Cooper, Sir A. on the thyroid gland.. 460
Cooper, Sir A. his case of imperf. fœtus 466
Corso completa di anatomia ........ 217
Coulson on diseases of the hip-joint.. 31
Cow-pox, natural, discovery of the .. 497
Craig, Mr. his case of spectral illusion 173
Crosse's retrospective address ...... 145
Croton oil in spleen .............. 486
Cure of epilepsy of 33 years' standing, 236
Curling on tetanus................. 55
Cutaneous tissue ................. 340
Cyclopædia of Anatomy & Physiology, 158
Cylindrical worms ................ 330
Cystine, case of by Mr. Bird ........ 256
Death ........................... 161
Death, two divisions of ............ 161
Deaths in London, table of ......... 358
Deformity of the pelvis ............ 485
Delirium of the dying............. 164
Deglutition ......... ........... 301
Dickson on fallacy of the art of physic, 135
Digestive organs, diseases of the .... 366
Digestive organs, affections of the.... 319
Digitalis a remedy for anasarca ...... 8
Dilated veins, operations on ........ 150
Disease of the hip-joint, Mr. Coulson on 31
Disease of the hip-joint, cause of .... 32
Disease, Dr. Carswell on ........... 297
Diseases, fatal, of London .......... 357
Diseases, fatal, at different ages...... 357
Diseases of infancy................ 364
Diseases of the digestive organs...... 366
Discovery of the natural cow-pox.... 497
Discussion on phrenology .......... 225
Dislocations, reduction of .. ....... 150
Dissecting-rooms of Paris .......... 423
Dissection of leg after tying iliac artery 552
Dissolution, approaching, signs of.... 164
Distortion of the foot from pressure.. 244
Diuretics, arrangement of the ...... 12
Donné on the properties of pus & blood 219
Double uterus, examples of ........ 223
Dreger, Dr. his case of tubal pregnancy 206
Dropsical effusion, elaterium a remedy, 9
Dropsy, nature and treatment of, by Dr. Seymour .................. 1
Dropsy of the abdomen ............ 18
Dublin, statistics of remedies in...... 186
Duchatelet on prostitution of Paris .. 49
Duchatelet on public hygiene.... 422, 537
Dupuytren ....................... 393
Dupuytren on the heart............ 567
Dynamometer.................... 150
Edward's case of cure of caries of the bones of the elbow ............. 286
Effects of concentrated malaria...... 369
Elaterium, its use in dropsy ........ 9
Electricity, medical uses of ......... 237
Elkington on the human skeleton .... 158
Empyema, case of ................. 514
Endemic fevers of the West Indies.... 368
Endocarditis, M. Cazeneuve on...... 513
Engrav. of arteries, by R. J. T. Knox, 158
Enlarged bursa over the patella...... 242
Enlargements of the spleen ......... 531
Entozoa in the human body ........ 147
Epidemic erysipelas ............... 550
Epilepsy of long standing, cure of.... 234
Epilepsy cured by nitrate of silver... 234
Erectile tissue.................... 337
Erysipelas ....................... 507
Evans on endemic fevers of West Indies 368
Evanson on management of children.. 362
Exaggerations of numbers of patients, 264
Examples of double uterus.......... 223
Exanthemata .................... 507
Excito-motory system ............ 300
Exostosis of the bones of the face.... 563
Extraordinary delivery ............ 151
Extra-uterine pregnancy, case of.... 207
Face, case of bony tumor of the .... 565
Factory system ................... 25
Factory statistics ................. 25
Factory system, working of the ..... 28
Fallacy of the art of physic, Dr. Dickson 135
False membranes, organization of.... 44
False aneurism of the brachial artery, 483
Family, a small one .............. 151
Farr's British Medical Almanack .... 156
Femur, dislocation of the head of the, 35
Fever the only disease ............. 136
Fever, use of tartar-emetic in ...... 551
Fever, thirst of .................. 551
Fevers of the West Indies, treatise on, 368
Fibrous and osseous tissues ........ 340
Flat worms...................... 329
Florence, medical museum at ....... 216
Fœtus, undecomposed, abortion of an, 187
Foot, distortion of the, from pressure, 244

Fracture of the thigh-bone ........ 542
France, midwifery in... .......... 563
Ganglia, office of the.............. 304
Ganglion, bunion, &c., Mr. Key on.. 241
Ganglion, division of the .......... 476
Gangrene of a serous membrane .... 48
Gangrene... ..................... 319
Geary, Mrs. N. her stays .......... 280
General knowledge, necessity of .... 375
Genius, difficulties of.............. 490
German contributions to practical medicine, by Dr. Thacker .......... 203
Glanders, case of ................ 500
Glosso-pharyngeal, Dr. Alcock on the, 474
Glosso-pharyngeal, Mr. Hilles on.... 474
Glosso-pharyngeal, division of the .. 475
Gustatory nerve, division of the .... 475
Gustatory & palatine nerves, division of 476
Guy's Hospital Reports............ 241
Graves' clinical lectures............ 550
Hahnemania ...................... 277
Hall on the nervous system ........ 297
Hamilton, Dr. his reclamation ...... 292
Head, changes in the.............. 488
Heart, Dr. Williams on palpitation of, 442
Heart, active palpitation of ........ 444
Heart, passive palpitation of........ 444
Heart, M. Dupuytren on the........ 567
Heart, traumatic compression of the 568
Heart, contusion of the............ 568
Heart, displacement of the ........ 568
Heygate on tic-douloureux ........ 154
Hilles, Mr. on Meckel's ganglion ... 474
Hip-joint, Coulson on disease of the.. 31
Hip-joint, cause of disease of the.... 32
Hip-joint, congenital displacement of 36
Hip-joint, treatment of disease of.... 38
Hip-joint, mode of "doing up" the.. 38
Hip-joint, nervous affections of the.. 415
Hodgkin, Dr. on the anatomy of the serous and mucous membranes.... 40
Hodgkin, Dr. on morbid anatomy.... 297
Hodgkin, Dr. on the Plancenta... .. 462
Homœopathy, Dr. Simpson on.. 137, 277
Homœopathy, Edwin Lee on........ 137
Homœopathy, Dr. Bureaud Riofrey on 137
Homœopathy, articles on in Med. Gaz. 137
Homœopathy, Mr. Crosse on...... 147
Hooping-cough, pathology of ...... 367
Horse, tetanus in the......... .... 63
Hospitals, statistics of ............ 261
Hospitals of the British legion ...... 574
How to become a crack physician 150 years ago...................... 287
Human body, on the weight of the .. 158
Hunchbacks, remarks on.......... 470
Hunter, his partnership with Cullen.. 447
Hunter, private habits of .......... 447
Hunter among the gods............ 491
Hunter a bad lecturer ............ 492
Hunter not a mere operator........ 493
Hunter's early rising.............. 493
Hunter's infirmity of temper........ 493
Hunterian oration ................ 445
Hunteriana....................... 489
Hydatids......................... 326
Hydatids in the lungs, case of ...... 555
Hydriodate of potass for ven. symptoms 188
Hydriodate of potass, properties of .. 479
Hydro-pneumato-pericardium, rare case of......................... 514
Hydrocele, on the cure of, by puncture 557
Hygiène publique ............ 422, 537
Hypogastric region, abscess in the .. 528
Hysteria ......................... 91
Hysteria followed by ischuria ...... 232
Hysterical affections, local.......... 415
Hysterical symptoms.............. 418
Illustrations of thoracic pathology .. 510
Illustrations of abdominal pathology 527
Imperfect fœtus, history of an...... 462
Imperfect fœtus, structure of an .... 463
Infancy, on the diseases of ........ 364
Infantile therapeutics.............. 365
Infantile asthma.. ................ 520
Inflammation of serous membranes.. 47
Influence of age on mortality........ 345
Influence of sex on mortality ...... 346
Influence of foreign station on army 349
Influence of opium, recovery from .. 560
Ingleby on obstetric medicine ...... 89
Injuries of the spinal cord.......... 300
Injuries of the spine, treatment of . 322
Insanity, four species of............ 266
Insanity, causes of................ 267
Insanity, statistics of...... ...... 356
Instrument for cure of artificial anus 179
Intermittent pleuropneumony ...... 370
Intermittent peritonitis............ 371
Intermittent irritation of the stomach 373
Intermittent fevers, M. Chomel on .. 506
Intoxication, recovery from the insensibility of ...................... 560
Inversion of the uterus..........99, 169
Iodine, semi-poisoning from ........ 201
Irish medical profession............ 585
Iritis, case of .................... 589
Irritation at the origins of nerves.... 405
Itch, the cause of all diseases . .... 141
Jaundice ........................ 131
Jaundice, Dr. Bright on............ 251
Jaundice, causes of................ 251
Jaundice, history of .............. 252
Jaundice, treatment of ............ 253
Jaundice, cases of ................ 254
Johnson, Mr. H. J. on ulceration of the cæcum .................... 197
Jugular vein, internal wound of the 175
Kennedy on apoplexy.............. 479
Key on ganglion, bunions, &c....... 241
Kidney, disease of, dependence of anasarca on ................ ... .. 16
King on the thyroid gland.......... 457
King on hydrocele ................ 550

Kissengen ........................ 87
Laryngismus stridulus, theory of.... 366
Lectures ........................ 383
Lee on continental mineral springs .. 84
Lee on homœopathy .............. 137
Leech-trade...................... 288
Lesions of the serous membranes.. .. 44
Lesions of the nervous system.. ..64, 222
Lesions of the muscular system .... 67
Lesions of the viscera, &c. ........ 68
Leucorrhœa ...................... 118
Lewis on hydrocele................ 558
Ligature of both carotid arteries, 149, 181
Ligature of the gluteal artery ...... 150
Ligature on both vertebral arteries.. 181
Ligature of the subclavian artery .. 504
Lithotripsy, Mr. Belinaye on........ 448
Liver, disease of the, a cause of ascites 18
Liver, A. Bonnet on disease of the .. 124
Liver, influence of mercury on the .. 134
Liver, affections of the............ 248
Liver, Dr. Addison on fatty degen. of 248
Liver, causes of fatty degeneration of 250
Liver, cases of abscess in the ...... 527
Liver, malignant diseases of the .... 553
Lobelia inflata in inflammations of the bronchial tubes ................ 586
Local hysterical affections.......... 415
Lombard, Dr. on typhus fever ...... 470
London University, charter of the.... 282
London, table of deaths in.......... 357
Lunatic asylum, Aberdeen.......... 265
Lungs, Weatherhead on disease of.... 441
Lungs, gout in the................ 441
Lungs, case of hydatids in the...... 555
M'Cabe on dis. connected with spiculæ 200
M'Culloch's statistics of British empire 345
M'Divitt on typhoid fever .......... 545
M'Divitt on poison of pork ........ 584
Malaria .......................... 368
Malaria, concentratrated, effects of .. 369
Malignant diseases of the liver ...... 553
Malposition of the stomach ........ 259
Manchester retrospective address .... 145
Manners of our ancestors . ........ 361
Marienbad ........................ 87
Maunsell on the diseases of children.. 362
Maxillary bone, removal of the...... 270
Mayo's human pathology .......... 297
Mayo's physiology ................ 297
Mechel's ganglion, Mr. Hilles on.... 474
Mechel's ganglion, use of ......... 477
Med. press, Mr. Crosse's remarks on 151
Medical professsion in France........ 210
Medical faculty of Paris, suppression of 212
Medical uses of electricity.......... 237
Medical statistics of the army ...... 269
Medical statistics, by Mr. Palmer.... 345
Medical education ................ 375
Medical study, Dr. Symonds on .... 375
Medical education, cheapness of .... 377
Medical philosophy, M. Bouillaud on 387
Medicine, German contributions to.. 203
Medicine in Chili, state of.......... 203
Membranes, serous & mucous, anat. of 40
Membranes, false ones, organization of 44
Membranes, serous, inflammation of 47
Mental cultivation of children ...... 363
Mercury in tetanus................ 72
Metastasis of neuralgia, case of...... 236
Midwifery in France .............. 563
Mineral springs of the continent.... 84
Mode of making stays ............ 280
Mode of medical study ............ 381
Moore's statistics of remedies in Dublin 186
Morbid anatomy, Dr. Hodgkin on .. 297
Morbid growths, cases of .......... 563
Morbility of the population ........ 353
Mortality, influence of age on ...... 345
Mortality, differences of, in various towns .......................... 347
Mortality, decrease of.............. 357
Mother's marks, mode of removing.. 209
Mucous tissue .................... 339
Muriate of Barytes................ 484
Muscles of the neck, spasms of the .. 247
Muscular spasms.................. 312
Museums, foreign, notice of........ 213
Museums of Strasbourg............ 215
Naples, cholera at ................ 505
Natural cowpox, discovery of the.... 497
Nature of angina pectoris .......... 522
Necessity of general knowledge...... 376
Negro, Tiedmann on the brain of the 467
Nerve, case of irritation of a........ 406
Nerves in connexion with tetanus.... 66
Nerves of sensation, affections of the 303
Nervous system, lesions of the....64, 232
Nervous system, Dr. Hall on the .... 297
Nervous affections, Sir B. Brodie on.. 403
Nervous affections, theory of...... .. 405
Nervous affections, treatment of .... 405
Nervous pains, character of ........ 410
Nervous pains, treatment of........ 413
Nervous affections, cases of ........ 413
Neuralgia of the spermatic nerves .. 234
Neuralgia ........................ 235
Neuralgia, case of metastasis of...... 236
New-born, convulsions of the ...... 480
Noble, Mr. his remarks on Dr. Alcock's experiments .................. 474
Norfolk and Norwich Hospital report 571
North London hospital ............ 270
Nosographie philosophique ........ 394
Notable discovery ................ 285
Nottingham hospital .............. 247
Numbers of patients and of deaths in the British hospitals ............ 262
Nux vomica, on the use of.......... 236
Obscure disease, case of............ 495
Obituary .......... ............ 289
Obstetric medicine, Mr. Ingleby on .. 89
Odds and ends.................... 150
Œsophagus, simulated stricture of .. 412

Old age, pneumonia of ............ 515
Operations, propriety of performing.. 276
Operator, danger of an ............ 148
Ophthalmic hospitals, state of ...... 569
Opium, use of, in tetanus .......... 73
Opium, influence of, recovery from .. 502
Organization of false membranes.... 44
Organology, M. Serres on some points 222
Origin of nerves, cases of irritation at 406
Original papers, list of ............ 453
Palmer, Mr. his life of Hunter...... 489
Palmer's medical statistics.......... 345
Paralysis of the voluntary muscles .. 311
Parasitical animals ................ 323
Paring a bottle nose .............. 541
Paris, prostitution of .............. 49
Parisian medical club.............. 286
Pathological anatomy .......... 40, 297
Pathology of tetanus.............. 64
Pathology, human, Mr. Mayo on .... 297
Pathology of the spinal cord, Sir. B. Brodie on .................. 297
Patients, exaggeration of numbers of 264
Pavia, medical museum at.......... 215
Pelvis, change which its bones undergo 33
Pelvis, deformity of the .......... 485
Pemphygus simplex .............. 486
Penis, new mode of amputating the.. 178
Penis, amputation of the .......... 487
Percussion, Dr. Williams on...... . 472
Percussion, force of the. .......... 473
Perforation of bowels, spontaneous, 530
Peritonitis, intermittent.......... . 371
Perry on contagious typhus ........ 481
Pharmaceutical works ............ 452
Phlebitis ...................... 289
Phrenology and apoplexy .......... 225
Physiology, Mr. Mayo on .......... 297
Pinel ........................ 393
Placenta, Dr. Hodgkin on the ...... 462
Pleuropneumonia ................ 508
Pleuropneumony, intermittent ...... 370
Pneumo-gastric nerves, ligatures on.. 183
Pneumonia, utility of bleeding in.... 142
Pneumonia ...................... 509
Pneumonia of old age ............ 515
Poisoning from colchicum.......... 239
Population, morbility of the........ 353
Pork, poison of .................. 584
Position of the thyroid gland ...... 459
Potass, hydriodate of.............. 188
Potass, hydriodate of, Mr. Wallace on 190
Pregnancy, signs of................ 100
Pregnancy, rare case of............ 208
Premature labour, induction of .... 94
Priapism ...................... 317
Professional study, mode of ........ 381
Professional study, spirit of ........ 385
Prolapsus uteri .................. 113
Prolapsus uteri, new truss for ...... 177
Properties of hydriodate of potash .. 479
Prostitutes, fecundity of .......... 49
Prostitutes, syphilis a disease of .... 49
Prostitution of Paris, M. Duchatelet on 49
Provincial Medical Journal ........ 288
Public Hygiene, M. Duchatelet on .. 537
Puncture for hydrocele ............ 557
Pupil, contraction of, in tetanus .... 57
Pus and blood, properties of........ 219
Pyrennees, sulphureous springs of the 85
Pyrexiæ, treatment of ............ 387
Pyrmont ........................ 86
Quain's anatomical engravings ...... 158
Rare case of pregnancy ............ 208
Reclamation of Dr. Hamilton ...... 292
Reclamations .................... 590
Removing of mother's marks, mode of 209
Respiration, affection of the ........ 314
Respiration in new-born infants, cause 525
Retraction of the tongue .. ...... 146
Retrospective address, by J. Crosse, Esq. 145
Rich and poor, statistics of ........ 549
Riofrey, Bureaud, on homœopathy .. 137
Saline thermal springs ............ 87
Scanty menstruation .............. 112
Science, encouragement of ........ 491
Scientific character, necessity of a .. 376
Scrofulous ulceration.............. 120
Seat of angina pectoris ............ 522
Semipoisons from iodine .......... 201
Sensation, affections of the nerves of 313
Sense of taste.................... 474
Sensorium, affection of the ........ 319
Serous membranes, morbid anatomy of 43
Serous membranes, lesions of the.... 44
Serous membranes, gangrene of a .. 48
Serous tissue .................... 338
Serres on some points in organology.. 222
Sewers of Paris .................. 537
Sex, influence of, on mortality...... 346
Seymour on dropsy................ 1
Sheppard, Mr. death of .......... .. 289
Sick poor, medical attendance on the 579
Simpson on homœopathy .......... 137
Skeleton, human, Mr. Elkington, on the 158
Skin, dropsy of the .... ......... 2
Skull, compound fracture of the .... 572
Solly on the brain ............ 158, 297
Spa .......................... 87
Spasms in tetanus ................ 57
Spasms of the muscles of the neck.. 247
Speculum, Dr. Balbirnie on the .... 105
Speculum, the apostle of the ...... 107
Speculum, mode of exploration with 110
Spectral illusions, Mr. Craig's case of 173
Spermatic nerves, neuralgia of the .. 234
Spiculæ, Dr. M'Cabe on diseases connected with.................... 200
Spillan's translation of the Clinique Medicale ...................... 40
Spinal chord, composition of the.... 468
Spinal apoplexy .................. 479
Spinal chord, pathology of the...... 298
Spinal chord, injuries of the........ 306

Spinal chord, concussion of ........ 308
Spinal chord, effects of injuries of the 310
Spine, wounds of the.............. 306
Spine, treatment of injuries of the .. 322
Spine, trephining the.............. 323
Spine, nervous affections of the .... 417
Spleen, enlargements of the......... 531
Spleen, arterial distribution of...... 531
Spleen mixture ................... 536
Spleen, croton oil in .............. 486
Spontaneous combustion .......... 500
Spontaneous combustion, origin of .. 502
Spontaneous combustion, cause of .. 503
State of the French medical profession 210
State of ophthalmic hospitals ...... 569
Statistical returns from hospitals, advantages of .................... 262
Statistics of factories.............. 25
Statistics of tetanus .............. 61
Statistics, medical, of the army.. 146, 269
Statistics of remedies in Dublin .... 186
Statistics of hospitals............... 261
Statistics of the British Empire .... 345
Statistics, Medical, Mr Palmer's .... 345
Statistics of rich and poor.......... 549
Stomach, malposition of the........ 259
Structure of the thyroid gland...... 457
Structure of an imperfect fœtus .... 464
Subclavian artery, ligature of the .. 504
Suicide, statistics of ... .......... 356
Symonds, Dr. on medical study .. . 375
Sympathetic system ............... 66
Sympathetic nerve, ligature on the .. 184
Syncope, various forms of.......... 163
System of Bichat ................ 390
Table of deviations from the normal state of an organ................ 42
Tæniæ............... ........ 329
Tarsus, alteration of the, from exercise 245
Taste, sense of .................. 474
Teachers, ordinance on recognition of 285
Tetanus, Mr. Curling on .......... 55
Tetanus, general account of ........ 56
Tetanus, contraction of the pupil in .. 57
Tetanus, spasms in................ 57
Tetanus, casue of death from........ 60
Tetanus, statistics of ............. 61
Tetanus, cause of ................ 62
Tetanus in the horse .............. 63
Tetanus, pathology of ............ 64
Tetanus, treatment of ............ 70
Tetanus, use of purgatives in ...... 72
Tetanus, various remedies in......... 72
Tetanus, use of tobacco in ........ 74
Tetanus, table of cases of ........ .. 78
Theory of nervous affections........ 405
Therapeutics of infancy .... ..... 365
Thigh-bone, fracture of the ........ 542
Thirst of fever ....... .......... 551
Thoracic pathology, illustrations of .. 510
Thorax, compression of, in pulmonary vomicæ ..................... 515
Thyroid gland, King on the ........ 45[illegible]
Thyroid gland, structure of ........ 45[illegible]
Thyroid gland, fluid of the.......... 458
Thyroid gland, position of the ...... 459
Thyroid gland, uses of the.......... 460
Thyroid gland, Sir A. Cooper on .... 460
Thyroid gland, of the cat.. ....... 460
Thyroid gland, of the dog.......... 461
Thyroid glands, of the sheep........ 461
Thyroid glands, of the horse........ 461
Thyroid gland, of the pig ......... 461
Thyroid gland, morbid anatomy of the 462
Tic douloureux, Dr. Heygate on .... 154
Tic douloureux, extraordinary case of 154
Tiedemann on the brain of the Negro 467
Tobacco a remedy in tetanus ...... 74
Tolerated brothels ........... .... 50
Tongue, retraction of the ........ . 146
Tongue, anatomy of the.. ......... 474
Transformations, analogous ........ 343
Transformations, fatty ........... 344
Travers on hydrocele .............. 559
Trephining the spine .............. 323
Tropical fevers, cause of............ 370
Trichinia spiralis.................... 331
Triple kidney in a man, case of.. ... 224
Truss for prolapsus uteri .......... 177
Tubal pregnancy, case of .......... 206
Tympanitis ....................... 419
Typhoid fever, Dr. M'Divitt on .... 545
Typhus fever, Dr. Lombard on ...... 471
Ulceration of the cæcum, case of .... 198
Ulnar artery, wound of the ....... 557
Umbilical cord ................. 543
Urinary organs, affections of the .... 317
Use of Meckel's ganglion ......... 477
Uterine hæmorrhage ............. 117
Uterus, retroversion of the ....... 93
Uterus, laceration of the ......... 96
Uterus, inversion of the....... 99, 169
Uterus, double, examples of ....... 223
Venereal diseases, hospitals for...... 88
Vertebral arteries, experiments on the 180
Vesicular worms ............... 326
Vichy ...................... 88
Vital statistics ............... 345
Wach on poisoning by baryta ...... 205
Warburton, Dr. works received from 217
Weatherhead on disease of the lungs.. 441
Williams on palpitation of the heart.. 442
Williams on percussion .......... 472
Womb, organic diseases of the.. .... 112
Working of the factory system ...... 28
Worms, vesicular ..... ........ 326
Wound of the internal jugular vein.. 175
Wound of the ulnar artery......... 557
Yelloly, Dr. on relief of the sick poor 579
Zarlenga, Dr. on cholera ......... 505

### NOTICES TO CORRESPONDENTS.

Several Articles have been postponed.—If C. D. will be kind enough to mention a place to which we may direct a Letter, we shall be most happy to communicate with him.

